# In Silico Comparative Analysis of Predicted B Cell Epitopes against Dengue Virus (Serotypes 1–4) Isolated from the Philippines

**DOI:** 10.3390/vaccines10081259

**Published:** 2022-08-05

**Authors:** Lyn Marielle I. Abesamis, Evan Gilles A. Aliping, Fritz Khrystian Gabriel H. Armada, Mirriam F. Danao, Pamela Denise B. del Valle, Zypher Jude G. Regencia, Emmanuel S. Baja, Antonio D. Ligsay

**Affiliations:** 1 Department of Biological Sciences, College of Science, University of Santo Tomas, España Blvd., Manila 1008, Philippines; 2 Department of Clinical Epidemiology, College of Medicine, University of the Philippines-Manila, Pedro Gil Street, Ermita, Manila 1000, Philippines; 3 Institute of Clinical Epidemiology, National Institutes of Health, University of the Philippines Manila, Pedro Gil Street, Ermita, Manila 1000, Philippines; 4 The Graduate School, University of Santo Tomas, España Blvd., Manila 1008, Philippines; 5 St. Luke’s Medical Center College of Medicine-William H. Quasha Memorial, 279 E Rodriguez Sr. Avenue, Quezon City 1112, Philippines

**Keywords:** dengue, epitope, peptide, B cell, DENV-1, DENV-2, DENV-3, DENV-4, vaccine

## Abstract

Dengue is a viral mosquito-borne disease that rapidly spreads in tropical and subtropical countries, including the Philippines. One of its most distinguishing characteristics is the ability of the Dengue Virus (DENV) to easily surpass the innate responses of the body, thus activating B cells of the adaptive immunity to produce virus-specific antibodies. Moreover, Dengvaxia^®^ is the only licensed vaccine for DENV, but recent studies showed that seronegative individuals become prone to increased disease severity and hospitalization. Owing to this limitation of the dengue vaccine, this study determined and compared consensus and unique B cell epitopes among each DENV (1–4) Philippine isolate to identify potential areas of interest for future vaccine studies and therapeutic developments. An in silico-based epitope prediction of forty (40) DENV 1–4 strains, each serotype represented by ten (10) sequences from The National Center for Biotechnology Information (NCBI), was conducted using Kolaskar and Tongaonkar antigenicity, Emini surface accessibility, and Parker hydrophilicity prediction in Immune Epitope Database (IEDB). Results showed that five (5) epitopes were consensus for DENV-1 with no detected unique epitope, one (1) consensus epitope for DENV-2 with two (2) unique epitopes, one (1) consensus epitope for DENV-3 plus two (2) unique epitopes, and two (2) consensus epitopes and one (1) unique epitope for DENV-4. The findings of this study would contribute to determining potential vaccine and diagnostic marker candidates for further research studies and immunological applications against DENV (1–4) Philippine isolates.

## 1. Introduction

In tropical and subtropical countries, the dengue virus (DENV) is recognized as one of the severe threats to public health [1]. In the Philippines, DENV can cause several illnesses but is frequently narrowed down to an acute flu-like disease that develops into a number of life-threatening complications [2]. According to the Epidemiology Bureau of the Philippine Department of Health (DOH), thousands of DENV cases have been recorded each year. The prevalence of DENV infection increased from an estimate of 46,300 in 2008 to 131,000 cases in 2017 [3]. Due to rainy seasons in the country, dengue outbreaks are significantly associated with this weather, increasing DENV infection cases [4].

The DENV naturally can avoid innate human response due to its evasive immune mechanism, ensuring viral entry and replication processes [5]. The adaptive human immune system manifests and responds to the infection through lymphocytes, particularly B cells and T cells, because the virion can easily exploit the innate immune system [6]. The B cells and T cells are vital components in achieving long-term immunity as they function for specific antibody production, antigen recognition, antigen destruction, and prevention of subsequent infections [7]. The immune response is induced by DENV infected antigen-presenting cells (APC) when the presented antigens are recognized by the cell’s receptor [8]. Furthermore, B cells activate and multiply into subtypes with specific functions once realized. The most notable of these are memory cells that can remember the antigen. This ability of the B cells to remember the previous infection lowers the chances of reinfection of the same serotype [9]. Due to DENV’s type-specificity, a problem may arise when the host is infected with DENV of a different serotype. The risk of secondary infection may still occur because previous DENV infection only provides partial immunity. The risk is attributed to the fact that DENV types are not too distinct as cross-reactive antibodies are produced [10]. In addition, the antibody (Ab)-dependent enhancement is another threat in secondary dengue infections. Ab enhancement occurs when a new DENV serotype infects the cell wherein the antibodies from the primary infection cannot neutralize the new type. The ab-virus complex is formed from these antibodies that further increase viral replication and enhance infection [11]. Because of the serotype-specificity and risks of Ab enhancement, the development of DENV vaccines is complex.

According to the World Health Organization (WHO), an ideal DENV vaccine should be given as a single dose. The vaccine should be effective in protecting against all four DENV serotypes, has long-term immunity, and bears no adverse effects [12]. The only approved DENV vaccine is the recombinant tetravalent dengue vaccine chimeric yellow fever virus (CYD-TDV or the Dengvaxia^®^), which functions by expressing the structural antigens of all four DENV serotypes [13,14]. However, CYD-TDV presented low efficacy against symptomatic dengue in all serotypes, especially DENV-1 and DENV-2, manifesting limited protection [15]. In addition, CYD-TDV is not recommended for seronegative individuals whose immune system is naive to any serotype of DENV since it acts as the primary subclinical infection. This problem potentially increases the severity of the secondary infection from a different serotype [14]. Therefore, the question of how both B cells can be effectively utilized to provide an optimal immunity against all DENV serotypes. Our research aims to compare and contrast the immunologic activities of B cells in the human immune system against the epitopes of the dengue virus serotypes (DENV 1–4) isolated from the Philippines. In this way, the optimal immunological response/s induced from B cells against each and all serotypes from the Philippines can be determined to identify potential candidate/s for further diagnostic and therapeutic research studies. This study focuses on determining consensus and unique B cell epitopes that can serve as areas of interest in local drug development.

## 2. Materials and Methods

### 2.1. Study Design

This study utilized an in silico approach focused on quantitative and qualitative systematic search and review research design. Data collection and interpretation were conducted from June to August 2021 through online databases, websites, and platforms, followed by an intensive comparative analysis from September 2021 to January 2022 using literature reviews and online repositories. These approaches provided a way to identify and juxtapose the physiochemical characteristics of B cells to elicit the optimal immunological response against the dengue virus serotypes (DENV 1–4) isolated from the Philippines.

### 2.2. Protocols and Equipment

The protocol used for the epitope prediction of B cells is generally based on a previous study that utilized in silico approach for the epitope prediction of DENV-2 [16]. Mainly, these methods adapted from the literature mentioned above are the collection of DENV strains from the National Center for Biotechnology Information (NCBI) from the United States National Library of Medicine and the Virus Pathogen Database Analysis and Resource (ViPR) from the United States National Institute of Allergy and Infectious Diseases. After searching from these databases, prediction of the B cell epitopes using the Immune Epitope Database (IEDB) and Analysis Resource then followed. Only linear epitopes were utilized in this study since these were the ones accessible in NCBI and ViPR which required DENV 1–4 protein sequences to be processed based on their respective sequence characteristics. Changes in their approach can be observed in the addition of DENV-1, -3, and -4 strains and the utilization of DENV (1–4) strains that have been isolated from the Philippines. These methods have also been guided by a previous study that tackled the fundamentals and methods for B cell epitope predictions [17].

#### 2.2.1. Collection of DENV Nucleotide Sequences

Ten (10) peptide sequences for each DENV (1–4) serotype were collected and sought from the NCBI (https://www.ncbi.nlm.nih.gov/ accessed on 26 December 2021) [18] and ViPR (https://www.viprbrc.org/ accessed on 26 December 2021) [19] databases through the use of their accession numbers. Out of the searched sequences on the databases, only envelope protein sequences obtained from the Philippines were considered and downloaded in FASTA format.

#### 2.2.2. Prediction and Documentation of B-Cell and T-Cell Epitopes

Each of the obtained DENV (1–4) sequences were utilized to analyze and calculate epitopes using the Epitope Prediction tools of the IEDB (https://www.iedb.org/ accessed on 26 December 2021) [20]. In addition, B cell epitopes were predicted based on their antigenicity, hydrophilicity and surface accessibility using Kolaskar and Tongaonkar Prediction, Parker Hydrophilicity Prediction, and Emini Surface Accessibility Prediction in IEDB, respectively. Findings were transferred to Microsoft Excel software for classification and sortation, where sequences were arranged from highest to lowest based on their respective threshold values and rankings. Furthermore, the frequency of each identified epitope on all sequences for all serotypes was also documented to identify consensus and unique ones.

### 2.3. Data Visualization and Analyses

The prediction tools for B cell epitopes calculated score values of sequences used for each parameter used. This prediction served as the basis for the arrangement and ranking of the peptides, where the first five (5) peptides with the highest threshold values were used. The threshold value was based on the provided value of the immunogenicity prediction tools in IEDB [21]. Notably, this guided the process of elimination sequences that have failed to reach the cut-off value. The predicted epitopes were then subjected to comparison and analysis with the results gathered from each serotype based on the records of literature reviews and online repositories. Once these findings have been cross-referenced with existing data and conducted studies, the immunogenic responses of B cell epitopes were compared and analyzed based on the available and aforementioned resources. Furthermore, the predicted residues were presented at a window size of 10. Window size generally refers to the number of amino acids wherein in this study, a window size of 10 was used to increase the number of overlaps present. Ideally. A 10–12 window size should be used for this type of studies to ensure specificity on the desired epitopes to be found [22]. In addition, the minimum amino acid sequence needed for proper folding of the discontinuous epitope in native proteins may range from 20 to 400 amino acids [23]. However, in our study the used a discontinuous linear segment, the number of amino acids may range from 6 to 12 in length [24].

## 3. Results

A total of forty (40) DENV (1–4) envelope protein sequences from Philippine isolates were successfully obtained from the NCBI and ViPR, wherein each DENV serotype was represented by ten (10) sequences. These envelope protein sequences were subsequently used for B cell epitope prediction using the analysis tools of IEDB. They were run for three (3) separate times to determine the predicted epitopes’ antigenicity, hydrophilicity, and accessibility scores with their corresponding threshold values.

### 3.1. Prediction of B Cell Epitopes

#### 3.1.1. DENV-1

Ten (10) envelope protein sequences from DENV-1 were analyzed, and amino acid sequences scored higher than the threshold value in Kolaskar and Tongaonkar antigenicity, Emini surface accessibility, and Parker hydrophilicity prediction tests in IEDB were selected. Table 1 shows the positions, peptide sequences, and mean scores of the top five 10 amino acid sequences. Results showed that the top epitopes among the 10 analyzed sequences were, all the same, indicating them as consensus epitopes. These were located at positions 242nd–51st (TAHAKKQEVV), 32nd–330th (LVQVKYEGTD), 141st–150th (VTVHTGDQHQ), 142nd–151st (TVHTGDQHQV), and 290th–299th (DKLTLKGVSY). Among these five (5) epitopes, the 242nd–251st contained the sequences with the highest antigenicity score (mean = 1.063), followed shortly by 321st–330th (mean = 1.062), then epitopes in the 141st–150th, 142nd–151st, and epitopes at 290th–299th having the same score (mean = 1.056). Interestingly, the ranking for these epitopes remained the same across all ten analyzed sequences, with epitope TAHAKKQEVV being the epitope at rank 1.

However, upon comparing the surface accessibility scores, the rankings were different. The 141st–150th and 142nd–151st epitopes scored the highest and had a similar score of 3.49, while the 242nd–251st came in third (mean = 2.93), followed by the 321st–330th (mean = 2.19), and 290th–299th (mean = 1.48), respectively. Meanwhile, in terms of hydrophilicity epitope, TAHAKKQEVV had the highest score (mean = 1.5561), followed by the 141st–150th and 142nd–151st, which had similar scores (mean = 1.2373). The epitopes LVQVKYEGTD and DKLTLKGVSY placed fourth (mean = 1.1965) and fifth (mean = 1.188), respectively. Notably, epitope TAHAKKQEVV exhibited the highest antigenicity score (mean = 1.063), while epitopes DKLTLKGVSY, VTVHTGDQHQ, and DKLTLKGVSY showed the lowest score (mean = 1.056).

DENV-1 was the only serotype to exhibit all top five (5) epitopes in all ten (10) analyzed sequences compared to other serotypes. Hence, all identified epitopes were determined as consensus, while no unique ones were observed among these sequences (see Supplementary Materials Table S1 for more details).

#### 3.1.2. DENV-2

For the B cell epitope prediction in DENV-2, ten (10) sequences were analyzed. Their threshold mean values ranged from 1.025 to 1.026 for antigenicity, 1.272 to 1.327 for hydrophilicity, and a score of 1 for accessibility (see Table 2). The results revealed one (1) consensus epitope that is consistent with all ten (10) sequences, which was PHAKKQDVVV (243rd–252nd). Throughout the analysis, this particular epitope repeatedly ranked first among all the epitopes, having a consistent antigenicity score (mean = 1.112) and hydrophilicity score (mean = 2.26), and an accessibility score ranging from 1.129 to 1.187. In addition, epitope QDKRFVCKHS (86th–95th) was also observed to be consistent in nine (9) sequences, ranking second in two (2) sequences, third in one (1) sequence, and fifth in six (6) sequences. Similarly, this epitope had persistent scores for antigenicity (mean = 1.062) and hydrophilicity (mean = 1.641) and varying scores for surface accessibility that ranged from 1.598 to 1.681.

Following this, epitope KHPATLRKYC (51st–60th) was frequently found among eight (8) of the analyzed sequences and ranked 4th in one (1) sequence and 2nd in the other seven (7) sequences. This epitope also had persistent antigenicity (mean = 1.07), hydrophilicity scores (mean = 1.74), and an accessibility score ranging from 1.869 to 1.966. Furthermore, epitopes KVVQPENLEY (128th–137th; antigenicity mean = 1.066, hydrophilicity mean = 1.79, accessibility scores range: 1.79 and 1.74) and VVQPENLEYT (129th–138th; antigenicity mean = 1.064, hydrophilicity mean = 1.74, accessibility scores range: 2.065 to 2.172) were both identified in the same six (6) sequences, repeatedly ranking 3rd and 4th in all of them. Another epitope that was present in four (4) of the ten (10) analyzed sequences was PIVTEKDSPV (356th–365th), which ranked 4th in three (3) sequences and 5th in one (1) sequence. This particular sequence obtained an antigenicity score of 1.061, a hydrophilicity score of 2.4, and a varying accessibility score that ranged from 1.181 to 1.197.

Moreover, epitope TLRKYCIEAK (55th–64th) was also identified in three (3) sequences, ranking 5th in each. This epitope had a predicted antigenicity score of 1.053, a hydrophilicity score of 1.3, and an accessibility score ranging from 1.109 to 1.113. Additionally, KQPATLRKYC (51st–60th) was an epitope that appeared in two (2) sequences, in which it ranked 3rd among all epitopes. Its antigenicity score was at 1.061, hydrophilicity at 2.13, and changing accessibility between 2.445 and 2.461. Notably, epitopes QDKRVVCKHS (86th–95th) and EQDKRVVCKH (85th–94th) were the two (2) epitopes observed to be unique among all sequences as they were found in one (1) and also the same sequence, and ranked 2nd and 3rd, sequentially. They exhibited antigenicity scores of 1.091 and 1.075, hydrophilicity scores of 3.42 and 3.55, and accessibility scores of 1.395 and 1.804, respectively.

In terms of the parameters, the highest values obtained for antigenicity (mean = 1.112), hydrophilicity (mean = 3.55), and accessibility (mean = 2.453) were presented by epitopes PHAKKQDVVV, EQDKRVVCKH, KQPATLRKYC, respectively. Conversely, all of their lowest values were exhibited by epitope TLRKYCIEAK, scoring 1.053 for antigenicity, 1.3 for hydrophilicity, and 1.113 for accessibility.

Ultimately, six (6) sequences with accession numbers BCG29750.1, BCG29751.1, BCG29752.1, AFN85177.1, AFN85178.1, and AOQ25641.1 revealed the same predicted epitopes in the same order—PHAKKQDVVV, KHPATLRKYC, KVVQPENLEY, VVQPENLEYT, and QDKRFVCKHS. Similarly, two (2) sequences with accession numbers AAR98806.1 and AAR98805.1 also exhibited the same predicted epitopes in the same order—PHAKKQDVVV, QDKRFVCKHS, KQPATLRKYC, PIVTEKDSPV, and TLRKYCIEAK. Additionally, one (1) particular sequence portrayed a mix of the epitopes mentioned above but in a different order—the sequence with accession number AOQ25658.1. As a result, the five (5) predicted epitopes of this sequence were identified to be PHAKKQDVVV, KHPATLRKYC, QDKRFVCKHS, PIVTEKDSPV, and TLRKYCIEAK. Finally, there was only one sequence that exhibited two unique epitopes, and this was the sequence with accession number AAR98804.1 with predicted epitopes as follows: PHAKKQDVVV, QDKRVVCKHS, EQDKRVVCKH, KHPATLRKYC, and PIVTEKDSPV (see Supplementary Materials Table S2 for more details).

#### 3.1.3. DENV-3

Ten (10) B cell epitopes of DENV-3 were predicted (see Table 3). In those analyses, the antigenicity threshold mean scores ranged from 1.023 to 1.026, the hydrophilicity mean scores ranged from 1.364 to 1.405, and the accessibility scores always resulted in 1.

Epitope YVCKHTYVDR (90th–99th) was the only consensus epitope, with a predicted antigenicity score of 1.118 in all ten (10) sequences. The said epitope also had a consistent hydrophilicity score of 1.74 in all sequences, but its accessibility score ranged from 1.039 to 1.07. Notably, the mean accessibility score of the consensus epitope was 1.048, which was relatively the lowest accessibility score among the predicted epitopes. Epitopes KVVQHENLKY (128th–137th), VVQHENLKYT (129th–138th), and VQHENLKYTV (130th–139th) were observed in nine (9) sequences and were usually found in the top 4 rankings next to YVCKHTYVDR (90th–99th). DENV-3 protein accession number QXI72689.1 was the only sequence devoid of the three epitopes mentioned above. Epitope PVVTKKEEPV (354th–363rd) was consistently found in eight (8) out of ten (10) sequences and had antigenicity and hydrophilicity scores of 1.075 and 2.53, respectively. Commonly found at fifth or sixth ranking, the said epitope had varying accessibility scores ranging from 1.889 to 1.945.

In all the DENV-3 envelope protein sequences analyzed, two to five of the described B cell epitopes were present. Two (2) protein sequences, particularly accession numbers AOQ25562.1 and AYP74620.1, were observed to have less-occurring epitopes PVVSKKEEPV (354th–363rd) and QHENLKYTVV (131st–140th), respectively. On the other hand, DENV-3 protein accession number QXI72689.1 had predicted epitopes DQNYVCKHTY (87th–96th), QDQNYVCKHT (86th–95th), and TVHTGDQHQV (142nd–151st) aside from YVCKHTYVDR (90th–99th) and PVVTKKEEPV (354th–363rd) (see Supplementary Materials Table S3 for more details).

#### 3.1.4. DENV-4

A total of ten (10) sequences were analyzed and studied for DENV-4, which revealed two (2) consensus epitopes consistent with all sequences, specifically PRSPSVEVKL and RSPSVEVKLP (see Table 4). These were noted to repeatedly appear within the top three (3) of each identified epitopes along 166th–175th and 167th–176th start and end, respectively, with a persistent antigenicity value of 1.082 and a hydrophilicity value of 1.83. Their accessibility values were observed to change for each sequence, despite the constant threshold value of 1.0 on all sequences, yet they displayed similar values on every sequence. Epitope HAKRQDVTVL found at 244th–253rd was also identified to be consistent among nine (9) sequences with rankings frequently seen along with the top four (4) to five (5). Such an epitope showed undeviating values for antigenicity and hydrophilicity at 1.078 and 1.87, respectively, despite varying values for accessibility.

Epitopes 169th–178th PSVEVKLPDY and 168th–177th SPSVEVKLPD that were observed within eight (8) of the sequences analyzed also followed. Both sequences portrayed consistent antigenicity and hydrophilicity values at 1.096 and 1.081, and 1.57 and 2.41, respectively. Notably, two (2) epitopes were also found to be unique among all ten (10) sequences, with 168th–177th SPSVEVKLPE and 170th–179th SVEVKLPEYG appearing only twice in two (2) sequences. Their antigenicity values were at 1.08 and 1.076, and hydrophilicity values were at 2.19 and 1.71, respectively. Furthermore, only one (1) epitope was unique among all sequences analyzed for DENV-4, the 366th–375th QHGTTVVKVK, residing along with the 5th spot of one (1) sequence. This epitope exhibited an antigenicity value of 1.082, 2.45 for hydrophilicity, and 1.001 for accessibility. Antigenicity threshold values ranged from 1.026 to 1.029, while hydrophilicity values ranged from 1.206 to 1.428, and accessibility remained at 1.0.

Of all the predicted epitopes, PSVEVKLPDY showed the highest value for antigenicity at 1.096, while the lowest value at 1.076 was seen in epitope SVEVKLPEYG. For hydrophilicity, the highest value was recorded at 2.45 due to the unique epitope, QHGTTVVKVK, and the lowest value at 1.57 by PSVEVKLPDF. Despite varying values in accessibility, the highest values were portrayed by consensus epitopes PRSPSVEVKL and RSPSVEVKLP, at 1.668, and the lowest value by the identified unique epitope, QHGTTVVKVK. Overall, five (5) sequences with accession numbers KJ946244.1, EU448458.1, MG895393.1, BCG29769.1, and ACC68759.1 revealed similar five (5) predicted epitopes. Such epitopes were identified to be PRSPSVEVKL, RSPSVEVKLP, PSVEVKLPDY, SPSVEVKLPD, and HAKRQDVTVL with minimal changes in order, particularly in that of BCG29769.1.

On the other hand, two (2) sequences with accession numbers KT750006.1 and AOQ25530.1 also exhibited similarly predicted epitopes of PRSPSVEVKL, RSPSVEVKLP, SPSVEVKLPE, HAKRQDVTVL, and SVEVKLPEYG in the same order. Furthermore, two (2) sequences with accession numbers JN575591.1 and ANC57623.1 also revealed similarly predicted epitopes of PSVEVKLPDY, PRSPSVEVKL, RSPSVEVKLP, SPSVEVKLPD, and HAKRQDVTVL in identical order. Meanwhile, one (1) sequence with accession number U18435.1 was the only sequence to exhibit a different set of predicted epitopes, particularly PSVEVKLPDY, PRSPSVEVKL, RSPSVEVKLP, QHGTTVVKVK, and SPSVEVKLPD (see Supplementary Materials Table S4 for more details).

## 4. Discussion

With all four (4) serotypes (DENV 1–4) circulating in the country for the last 50 years, DENV has been considered an endemic disease in the Philippines [25]. Currently, the DENV-1 is recognized as the most predominant type of serotype reported by the DOH, overtaking DENV-2, which was the most isolated serotype from 1995 to 2010. However, a reported shift of predominance of the DENV-3 serotype [26]. In addition, DENV-3 had affected over 64% of the total cases from January to November 2019, when the DOH declared a national dengue epidemic [3]. Therefore, the co-circulation of all serotypes in the country with varying dominance sufficiently calls for a multi-faceted approach to combat such infection.

This study used three (3) parameters to determine the DENV (1–4) epitopes that can induce the optimal immunological response: antigenicity, surface accessibility, and hydrophilicity. These characteristics are known to be responsible for the immunogenicity of peptides. Furthermore, they are often defined based on the frequencies and positions of specific amino acids associated with B cell receptors, paratopes situated on variable regions of antibodies, and the innate aqueous environment of the host [16]. Specifically, DENV triggers B cell activation to produce virus-specific antibodies such as immunoglobulins I, G, and A (IgM, IgG, and IgA). A part of this eventually binds to the viral envelope protein (E protein), the most antigenic part of the virus, before neutralizing them to hinder their entry into target host cells [27].

This study identified the top peptides of the DENV-1 serotype with the highest antigenicity scores. In addition, this study has revealed consensus amino acid sequences present in the ten isolates, which have also topped the rankings in terms of antigenicity. Specifically, the epitope found at 242nd and 251st (TAHAKKQEVV) is the peptide with the highest scores in the antigenicity test in all isolates for DENV-1. The congruency of these results can be brought by the presence of a single DENV-1 genotype that dominates the country [25]. Phylogenetic analysis and genotypic identification have shown that only one (1) genotype (IV) of DENV-1 has circulated in the country since 1974. A previous strongly considered the Philippines as its point of origin and probably the source of its introduction to other Pacific and Asian countries [28].

In comparison, co-circulation of at least two (2) genotypes (I and II) have been observed in several other Asian countries [29,30]. Archipelagic topography plays a significant factor in the persistence of genotype IV in the country, which can explain the congruence of the results in which all peptide sequences are present in the isolates [31]. In addition, in a previous study, specific DENV-1 crystal structures have been identified to bind to epitopes situated along EDIII that dictates the neutralization process depending on the genotype of such serotype [32]. This structure shows such a region as an ideal area of interest for further studies to better analyze the induction of antibodies during the infection.

Interestingly, our results have shown that epitope TVHTGDQHQV is present in DENV-1 and DENV-3. Both predicted peptides are found in the same position (142nd and 141st) and have similar antigenicity scores in both serotypes but differ in surface accessibility and hydrophilicity. Although DENV serotypes are genetically distinct, one study has revealed that this diversity is only between 20–30%, which may explain why epitope TVHTGDQHQV is present in DENV-1 and DENV-3 [30]. Such findings have also been supported by an earlier study where researchers have detected the presence of TVHTGDQHQV on their B cell epitopes prediction of DENV-3, further noting through a 3D model structure that the peptide is fully projected on the surface [15]. Furthermore, in a study conducted on rodents, a murine monoclonal antibody (mAb) was observed to bind to EDIII from DENV-1 through DENV-3 [33]. This experiment showed that the binding process has successfully prevented cell attachment due to the disrupted virion architecture of DENV, thus proving similarities between the two serotypes [33].

Another observation worth noting is the presence of epitopes from different envelope gene sequences with the same position (start and end) but not precisely the same peptide sequence. The results obtained from the prediction of B cell epitopes from DENV-2 showed that gene sequences AAR98806.1 and AAR98805.1 have the epitope KQPATLRKYC, while gene sequences AAR98804.1, BCG29750.1, BCG29751.1, BCG29752.1, AFN85177.1, AFN85178.1, AOQ25641.1, and AOQ25658.1 have the epitope KHPATLRKYC. These two epitopes start from 51st and end with 60th; however, only one letter in their peptide sequence is different. This observation can also be seen with the epitopes QDKRFVCKHS and QDKRVVCKHS, which start from 86th and end with 95th. However, the latter epitope is found only in gene sequence AAR98804.1, while the former is found in every gene sequence except AAR98804.1. This particular incident may be attributable to the capability of DENV-2 to exhibit different morphologies and have structural changes, causing it to have other antigenic properties [34,35]. Moreover, some strains of both DENV-1 and DENV-2 and the Zika virus can all transform into club-shaped particles, a conformation different from their original structure to possibly evade treatment and vaccines, as reported by an earlier study [36].

Due to the ability of DENV-2 to mutate and morph, this ability allows this serotype to bypass the human host’s immune system in some cases, which poses a complication to the development of vaccines and therapeutics [37]. This ability of the DENV-2 to mutate and morph is also the reason why the only licensed tetravalent dengue vaccine, Dengvaxia^®^ (CYD-TDV), has shown lower efficacy against DENV-2 than the other serotypes [38]. Similar findings have also been published in a previous study which reported that CYD-TDV is 50% to 80% efficacious against DENV-1,-3,-4, but only 35% to 42% against DENV-2 [39], which is found to be the most virulent and frequently associated with more severe dengue cases among all serotypes [40]. For this reason, it is crucial to determine its dominant target for dengue virus therapeutic studies.

Researchers have mapped the epitopes of strongly neutralizing human mAb using human dengue virus polyclonal immune serum and recombinant dengue viruses [33]. In addition, they have found that the primary target of DENV-2 neutralizing bodies is the epitopes that belong to EDIII, as evidenced by the DENV-2-infected individuals in their study that produced type-specific antibodies that target the same region. This finding is consistent with a previous study that reported that targeting antibodies to the type-specific antigenic site on EDIII is the best for DENV-2 [41]. Although in some cases, at least two (2) neutralizing epitopes are targeted disproportionately following the initial DENV-2 infections, and this is attributable to the instances where neutralization titers do not always track with EDIII epitopes. Gallichotte et al. (2018) then discovered that EDI epitope is the second dominant target domain as the study showed strong neutralizing activities against this region as well; however, the overall response is still higher with antibodies tracking with epitopes along EDIII [39,41].

Notably, predicted epitopes in DENV-2, such as QDKRFVCKHS and QDKRVVCKHS of DENV-2, appeared closely similar to that of QDQNYVCKHT from DENV-3 at the 86th–95th start and end. Similar observations are also recorded on DENV-2 epitopes, KVVQPENLEY and VVQPENLEYT, and DENV-3 epitopes, KVVQHENLKY and VVQHENLYYT, respectively. Despite being situated along with similar positions, such significant similarities and minor differences between amino acid orders can be attributed to similarities in both serotypes, such as their effect on the host’s interferon-gamma production (IFN-γ) during infection. Previous research showed that IFN-γ-induced nitric oxide production induced during DENV-2 infection leads to host protection during DENV-3 infection of the same patient [42]. Such occurrence leads to host resistance to secondary infection brought by a different serotype, DENV-3, explicitly due to the effect of IFN-γ in reducing viral replication to prevent severe disease manifestation and lethality. In addition, nonstructural protein 5 (NS5) proteins generated via N-terminal ubiquitin cleavage for both serotypes accumulate in similar locations, particularly in the nucleus of host cells [43].

Among the serotypes subjected to IEDB analyses, DENV-3 had a total of 10 epitopes predicted. This relatively high number of epitopes may be attributed to the presence of two genotypes (I and II) of DENV-3. In a study on the genotypic persistence of DENV in the Philippines, a previous report described the lineage of genotype I as the predominating sequence since 1980s, while a co-circulating genotype II subsequently emerged and became a minor population [25]. Moreover, the genotypic variation of envelope glycoproteins within each serotype varies by 6–9% [44]. This variation further explains the frequent occurrence of several epitopes in DENV-3 protein sequences. The human antibody response against DENV-3 is still less studied than the other serotypes. Identification of DENV-3 antigenic sites from a previous report describes the neutralizing antibodies of DENV-3 as complex and diverse [45]. The study further noted the presence of epitopes clustering in antigenic sites primarily located in EDs I and II. In silico approaches for tetravalent vaccines using DENV-2 and DENV-3 have also been established beforehand. The said serotypes can be used as backbones for the design due to their high predominance in South East Asian regions and the E proteins are commonly utilized due to their function as attachments on the host cell surface [46].

Interestingly, predicted epitopes in DENV-4, specifically PRSPSVEVKL, RSPSVEVKLP, PSVEVKLPDY, SPSVEVKLPD, SPSVEVKLPE, and SVEVKLPEYG appear to be similar to identified epitopes in the findings of a recent study [44]. Such epitopes are the following: PSVEVKLPDYGELTL, TATITPRSPSVEVKL, TPRSPSVEVKLPDYG, and VKLPDYGELTLDCEP, which exhibited minimal differences in amino acid order. Their study states that these discovered epitopes elicit strong flavivirus cross-reactive antibodies during both primary and secondary infections and are concentrated along with different structures, such as the capsid, E, NS1, NS3, and NS5 proteins. In addition, unique epitopes and epitope variants are also found to increase during secondary infection, thus indicating such areas as epitopes inducing optimal immunological responses [47].

DENV-4 is considered the least studied serotype of DENV despite its circulation in East Asia and Southeast Asia due to its relatively mild clinical manifestations [48]. It comprises roughly 70% conserved E protein regions with highly divergent sequences and no variations, similar to that of other serotypes. Upon contact with Dengvaxia^®^ it produces the highest levels of serotype-specific neutralizing antibodies among all serotypes, thus indicating a high vaccine efficacy against DENV-4 infection of naive individuals [49]. This high vaccine efficacy is due to the large number of components of the vaccine that attach to the quaternary structures of DENV-4, thereby almost acting as a monovalent vaccine against this serotype [50]. Moreover, in a previous study, it was found that surface envelope molecules of DENV-4 are more tightly packed as compared to that of other serotypes, thereby contributing to its rigidness and stability, unlike that of DENV-1,-2, and -3 [51].

Furthermore, a previous report also discovered that monoclonal antibodies from DENV-4-infected individuals with epitopes located around EDI and II hinge regions were the designated targets of DENV-4’s neutralizing antibodies [49]. These findings are observed via memory B cells and even long-lived plasma cells. Such regions are critical in conformational changes that the E protein encounters during low pH upon binding to the endosomal membrane for its viral uncoating. Moreover, viral RNA can be released into the cellular cytoplasm of the host to initiate infection [52]. Similar findings have also been observed in an earlier study that isolated a monoclonal antibody from a DENV-4-infected chimpanzee to observe substantial neutralizing activities directed to the EDI region [30]. This observation makes epitopes located along such regions ideal areas of interest for therapeutic studies and the progress of DENV vaccines. This is brought by their ability to hinder conformational changes in the E protein to disrupt the fusion, entry, and initiation of infection, as well as the increased possibility of the epitopes existing on different genotypes of DENV-4 [52].

While this study focuses on in silico approaches to identify dengue virus epitopes that induce the optimal immunological response/s from both B cells, it is subjected to potential biases rooted in the vastness of the available DENV strains in the database from different dengue-endemic countries. Such strains, which may have different epitope sequences, may also stimulate different immunological responses from B cells. To resolve and minimize the effect of the bias mentioned above, we narrowed down the locale of the study by focusing on Philippine isolates. The study also revolved around B cell immune responses alone. In addition, the availability of existing literature reviews and records from online databases tackling DENV epitopes may not also correlate to the gathered data. Specific keywords explicitly related to the subject matter were used to resolve this, such as dengue virus (DENV), B-cell epitope, epitope prediction, Philippines, and immunological response.

To our knowledge, this is the first in silico study conducted on the four DENV serotypes from Philippine isolates. Understanding and identifying the mechanism of actions of the B cells against each DENV serotype during primary and secondary infections would significantly contribute to the current knowledge on the matter and is the first step in widening the public’s access to further research studies and immunological applications.

In general, epitopes are used in vaccine design and development as a means of determining short amino acid sequences of a specific protein that can elicit a more direct and potent immune response for the body [53]. Such an approach is different from traditional processes of vaccine design and development that have been used in the past, making epitope vaccines a fast and straightforward measure. In this study, relevant antigen epitopes have been determined through an immunoinformatics approach and computational techniques, as this is an in silico study. These results could be used and applied in succeeding in vitro experiments to further quantify the epitopes’ antigenic, hydrophilic, and accessible properties. An example of which is through immunizing animal models to determine the immunogenicity of the peptide. Immunological assays such as enzyme-linked immunosorbent assay (ELISA) are then used to monitor and measure the antibody production. Thus, this study provides potent epitopes that can become potential vaccine candidates when further studies are performed. Our research findings could be also highly significant and beneficial as they will present the public with ample knowledge of the DENV serotypes within the local setting and aid health-allied professionals to determine the appropriate approach to ministering to individuals afflicted with DENV infection. Furthermore, our result may benefit researchers with answers concerning the DENV and the human immune response and enable future researchers to gain the necessary conclusion and formulate appropriate recommendations for future studies to come with respect to their findings.

There are also limitations in this study and we acknowledge them. This study is only focused on identifying the epitopes common in all four dengue virus serotypes (DENV 1–4) that will cause the optimal immunological responses from B cells. As it is limited to in silico approaches, this study did not include immunodiagnostic methods involving in vivo tests and assays such as the ELISA, Western blotting, and the like; which may further expand the understanding of the actions of the B cells against each DENV serotypes. In vivo testing of the predicted B cell epitopes of all DENV (1–4) serotypes in this study is highly recommended. This further testing will enable epitopes to be clinically measured and evaluated for their potential immunogenicity and therapeutic properties with regard to developing an effective monovalent or multivalent vaccine that specifically targets Philippine isolates of the virus.

Moreover, a more comprehensive range of isolates used for epitope prediction may reveal new findings or support the presented hypothesis of the study, particularly consensus and unique epitopes. Lastly, a comparative analysis with T cell epitopes predicted from the identical DENV isolates is a subject of interest that may provide a robust framework for vaccine development and future DENV studies. Studies investigating T cell response to DENV is well established [54,55,56,57,58]. These studies demonstrated the pathological and protective function of T cells against DENV infection. Specifically, both CD4 T cells and CD8 T cells play an immunoprotective role against the pathogen. To elicit a T cell immune response, the epitope bound to the MHC molecule must be recognized by the receptors present in the T cells. Once recognized, signals are sent for the release of perforin and granzymes that invade the pores of the infected cell to induce cell death [59]. Research had also identified DENV proteins that elicit T cell immune response, specifically NS3, Capsin, NS5, and NS4A/B proteins are the primary response targets for CD8 T cells [54]. Among these, NS3 was considered to be the most frequent target that induces CD8 T cell response. Meanwhile, capsid is the most preferred target for CD4 T cells, followed by Envelope, NS3, NS2A/B and NS5 proteins [60]. The relationship between B cells and T cells in response to DENV infections can give more knowledge and understanding of the adaptive immunological response of the human body as a host.

## Figures and Tables

**Table 1:** Antigenicity, surface accessibility, hydrophilicity, and frequencies of top epitopes of DENV-1 E protein.

Position		Peptide Sequence	Antigenicity (mean)	Surface Accessibility (mean)	Hydrophilicity (mean)	Frequency (*n* = 10)	Rank According to Antigenicity
Start	End						
242	251	TAHAKKQEVV	1.063	2.93	1.5561	10	1
321	330	LVQVKYEGTD	1.062	2.19	1.1965	10	2
141	150	VTVHTGDQHQ	1.056	3.49	1.2373	10	3
142	151	TVHTGDQHQV	1.056	3.49	1.2373	10	4
290	299	DKLTLKGVSY	1.056	1.48	1.188	10	5

**Table 2:** Antigenicity, surface accessibility, hydrophilicity, and frequencies of top epitopes of DENV-2 E protein.

Position		Peptide Sequence	Antigenicity (mean)	Surface Accessibility (mean)	Hydrophilicity (mean)	Frequency (*n* = 10)	Rank According to Antigenicity
Start	End						
243	252	PHAKKQDVVV	1.112	1.158	2.26	10	1
86	95	QDKRVVCKHS	1.091	1.395	3.42	1	2
85	94	EQDKRVVCKH	1.075	1.803	3.55	1	3
51	60	KHPATLRKYC	1.07	1.916	1.74	8	4
128	137	KVVQPENLEY	1.066	2.116	1.79	6	5
129	138	VVQPENLEYT	1.064	1.527	1.74	6	6
86	95	QDKRFVCKHS	1.062	1.641	2.87	9	7
51	60	KQPATLRKYC	1.061	2.453	2.13	2	8
356	365	PIVTEKDSPV	1.061	1.193	2.4	4	9
55	64	TLRKYCIEAK	1.053	1.113	1.3	3	10

**Table 3:** Antigenicity, surface accessibility, hydrophilicity, and frequencies of top epitopes of DENV-3 E protein.

Position		Peptide Sequence	Antigenicity (mean)	Surface Accessibility (mean)	Hydrophilicity (mean)	Frequency (*n* = 10)	Rank According to Antigenicity
Start	End						
90	99	YVCKHTYVDR	1.118	1.048	1.74	10	1
354	363	PVVSKKEEPV	1.085	1.763	2.66	1	2
128	137	KVVQHENLKY	1.078	2.102	1.58	9	3
129	138	VVQHENLKYT	1.076	1.517	1.53	9	4
130	139	VQHENLKYTV	1.076	1.517	1.53	9	5
131	140	QHENLKYTVV	1.076	1.508	1.53	1	6
354	363	PVVTKKEEPV	1.075	1.907	2.53	8	7
87	96	DQNYVCKHTY	1.072	2.023	2.99	1	8
86	95	QDQNYVCKHT	1.057	2.235	3.78	1	9
142	151	TVHTGDQHQV	1.056	1.194	3.49	1	10

**Table 4:** Antigenicity, surface accessibility, hydrophilicity, and frequencies of top epitopes of DENV-4 E protein.

Position		Peptide Sequence	Antigenicity (mean)	Surface Accessibility (mean)	Hydrophilicity (mean)	Frequency (*n* = 10)	Rank According to Antigenicity
Start	End						
169	178	PSVEVKLPDY	1.096	1.558	1.57	8	1
166	175	PRSPSVEVKL	1.082	1.559	1.83	10	2
167	176	RSPSVEVKLP	1.082	1.559	1.83	10	3
366	375	QHGTTVVKVK	1.082	1.001	2.45	1	4
168	177	SPSVEVKLPD	1.081	1.332	2.41	8	5
166	177	SPSVEVKLPE	1.08	1.366	2.19	2	6
244	253	HAKRQDVTVL	1.078	1.193	1.87	9	7
170	179	SVEVKLPEYG	1.076	1.022	1.71	2	8

## Data Availability

The data are available upon request to the corresponding author.

## Supplementary Materials

The following supporting information can be downloaded at: https://www.mdpi.com/article/10.3390/vaccines10081259/s1, Table S1: Antigenicity, surface accessibility, and hydrophilicity of epitopes of DENV-1 E protein; Table S2: Antigenicity, surface accessibility, and hydrophilicity of epitopes of DENV-2 E protein; Table S3: Antigenicity, surface accessibility, and hydrophilicity of epitopes of DENV-3 E protein; Table S4: Antigenicity, surface accessibility, and hydrophilicity of epitopes of DENV-4 E protein.
