# Supplementary material for: In Silico Comparative Analysis of Predicted B Cell Epitopes against Dengue Virus (Serotypes 1–4) Isolated from the Philippines
Source: Vaccines (Basel). 2022 Aug 5;10(8):1259. doi: 10.3390/vaccines10081259 (PMC9415047; doi:10.3390/vaccines10081259)
Supplement: Supplementary file 1 [file vaccines-10-01259-s001.zip › vaccines-1856909-supplementary.pdf]

Supplementary Material

Table S1. Antigenicity, surface accessibility, and hydrophilicity of epitopes of DENV-1 E protein.

GenBank Accession No.	Position			Antigenicity	Hydrophilicity	Surface accessibility	Rank according to antigenicity
AAR01106.1	Start	End	Peptide Sequence	Cut-off = 1.029	Cut-off = 1.402	Cut-off = 1	
	242	251	TAHAKKQEVV	1.063	2.93	1.58	1
	321	330	LVQVKYEGTD	1.062	2.19	1.215	2
	141	150	VTVHTGDQHQ	1.056	3.49	1.257	3
	142	151	TVHTGDQHQV	1.056	3.49	1.257	4
	290	299	DKLTLKGVSY	1.056	1.48	1.207	5
**AAR01107.1**				**Cut-off = 1.029**	**Cut-off = 1.41**	**Cut-off = 1**	
	242	251	TAHAKKQEVV	1.063	2.93	1.54	1
	321	330	LVQVKYEGTD	1.062	2.19	1.184	2
	141	150	VTVHTGDQHQ	1.056	3.49	1.225	3
	142	151	TVHTGDQHQV	1.056	3.49	1.225	4
	290	299	DKLTLKGVSY	1.056	1.48	1.176	5
**AAR01108.1**				**Cut-off = 1.03**	**Cut-off = 1.404**	**Cut-off = 1**	
	242	251	TAHAKKQEVV	1.063	2.93	1.546	1
	321	330	LVQVKYEGTD	1.062	2.19	1.189	2
	141	150	VTVHTGDQHQ	1.056	3.49	1.229	3
	142	151	TVHTGDQHQV	1.056	3.49	1.229	4
	290	299	DKLTLKGVSY	1.056	1.48	1.18	5
**AAR01109.1**				**Cut-off = 1.029**	**Cut-off = 1.412**	**Cut-off = 1**	
	242	251	TAHAKKQEVV	1.063	2.93	1.557	1
	321	330	LVQVKYEGTD	1.062	2.19	1.197	2
	141	150	VTVHTGDQHQ	1.056	3.49	1.238	3
	142	151	TVHTGDQHQV	1.056	3.49	1.238	4
	290	299	DKLTLKGVSY	1.056	1.48	1.189	5
**AAR01110.1**				**Cut-off = 1.03**	**Cut-off = 1.385**	**Cut-off = 1**	

	242	251	TAHAKKQEVV	1.063	2.93	1.555	1
	321	330	LVQVKYEGTD	1.062	2.19	1.196	2
	141	150	VTVHTGDQHQ	1.056	3.49	1.236	3
	142	151	TVHTGDQHQV	1.056	3.49	1.236	4
	290	299	DKLTLKGVSY	1.056	1.48	1.187	5
**AAR01111.1**				**Cut-off = 1.03**	**Cut-off = 1.404**	**Cut-off = 1**	
	242	251	TAHAKKQEVV	1.063	2.93	1.556	1
	321	330	LVQVKYEGTD	1.062	2.19	1.196	2
	141	150	VTVHTGDQHQ	1.056	3.49	1.237	3
	142	151	TVHTGDQHQV	1.056	3.49	1.237	4
	290	299	DKLTLKGVSY	1.056	1.48	1.188	5
**QMT58614.1**				**Cut-off = 1.03**	**Cut-off = 1.404**	**Cut-off = 1**	
	242	251	TAHAKKQEVV	1.063	2.93	1.557	1
	321	330	LVQVKYEGTD	1.062	2.19	1.197	2
	141	150	VTVHTGDQHQ	1.056	3.49	1.238	3
	142	151	TVHTGDQHQV	1.056	3.49	1.238	4
	290	299	DKLTLKGVSY	1.056	1.48	1.188	5
**QMT58617.1**				**Cut-off = 1.03**	**Cut-off = 1.404**	**Cut-off = 1**	
	242	251	TAHAKKQEVV	1.063	2.93	1.556	1
	321	330	LVQVKYEGTD	1.062	2.19	1.196	2
	141	150	VTVHTGDQHQ	1.056	3.49	1.237	3
	142	151	TVHTGDQHQV	1.056	3.49	1.237	4
	290	299	DKLTLKGVSY	1.056	1.48	1.188	5
**BCG29720.1**				**Cut-off = 1.03**	**Cut-off = 1.379**	**Cut-off = 1**	
	242	251	TAHAKKQEVV	1.063	2.93	1.562	1
	321	330	LVQVKYEGTD	1.062	2.19	1.201	2
	141	150	VTVHTGDQHQ	1.056	3.49	1.242	3
	142	151	TVHTGDQHQV	1.056	3.49	1.242	4
	290	299	DKLTLKGVSY	1.056	1.48	1.192	5
**BCG29747.1**				**Cut-off**	**Cut-off**	**Cut-off = 1**	

				= 1.03	= 1.402		
	242	251	TAHAKKQEVV	1.063	2.93	1.552	1
	321	330	LVQVKYEGTD	1.062	2.19	1.194	2
	141	150	VTVHTGDQHQ	1.056	3.49	1.234	3
	142	151	TVHTGDQHQV	1.056	3.49	1.234	4
	290	299	DKLTLKGVSY	1.056	1.48	1.185	5

Table S2. Antigenicity, surface accessibility, and hydrophilicity of epitopes of DENV-2 E protein.

GenBank Accession No.	Position			Antigenicity	Hydrophilicity	Surface accessibility	Rank according to antigenicity
**AAR98806.1**	**Start**	**End**	**Peptide Sequence**	**Cut-off = 1.025**	**Cut-off = 1.285**	**Cut-off = 1**	
	243	252	PHAKKQDVVV	1.112	2.26	1.168	1
	86	95	QDKRFVCKHS	1.062	2.87	1.653	2
	51	60	KQPATLRKYC	1.061	2.13	2.461	3
	356	365	PIVTEKDSPV	1.061	2.4	1.2	4
	55	64	TLRKYCIEAK	1.053	1.3	1.116	5
**AAR98805.1**				**Cut-off = 1.025**	**Cut-off = 1.272**	**Cut-off = 1**	
	243	252	PHAKKQDVVV	1.112	2.26	1.16	1
	86	95	QDKRFVCKHS	1.062	2.87	1.643	2
	51	60	KQPATLRKYC	1.061	2.13	2.445	3
	356	365	PIVTEKDSPV	1.061	2.4	1.192	4
	55	64	TLRKYCIEAK	1.053	1.3	1.109	5
**AAR98804.1**				**Cut-off = 1.025**	**Cut-off = 1.327**	**Cut-off = 1**	
	243	252	PHAKKQDVVV	1.112	2.26	1.15	1
	86	95	QDKRVVCKHS	1.091	3.42	1.395	2
	85	94	EQDKRVVCKH	1.075	3.55	1.803	3
	51	60	KHPATLRKYC	1.07	1.74	1.904	4
	356	365	PIVTEKDSPV	1.061	2.4	1.181	5
**BCG29750.1**				**Cut-off = 1.026**	**Cut-off = 1.304**	**Cut-off = 1**	

	243	252	PHAKKQDVVV	1.112	2.26	1.172	1
	51	60	KHPATLRKYC	1.07	1.74	1.941	2
	128	137	KVVQPENLEY	1.066	1.79	2.144	3
	129	138	VVQPENLEYT	1.064	1.74	1.547	4
	86	95	QDKRFVCKHS	1.062	2.87	1.659	5
**BCG29751.1**				**Cut-off = 1.025**	**Cut-off = 1.326**	**Cut-off = 1**	
	243	252	PHAKKQDVVV	1.112	2.26	1.129	1
	51	60	KHPATLRKYC	1.07	1.74	1.869	2
	128	137	KVVQPENLEY	1.066	1.79	2.065	3
	129	138	VVQPENLEYT	1.064	1.74	1.49	4
	86	95	QDKRFVCKHS	1.062	2.87	1.598	5
**BCG29752.1**				**Cut-off = 1.026**	**Cut-off = 1.297**	**Cut-off = 1**	
	243	252	PHAKKQDVVV	1.112	2.26	1.187	1
	51	60	KHPATLRKYC	1.07	1.74	1.966	2
	128	137	KVVQPENLEY	1.066	1.79	2.172	3
	129	138	VVQPENLEYT	1.064	1.74	1.567	4
	86	95	QDKRFVCKHS	1.062	2.87	1.681	5
**AFN85177.1**				**Cut-off = 1.025**	**Cut-off = 1.325**	**Cut-off = 1**	
	243	252	PHAKKQDVVV	1.112	2.26	1.141	1
	51	60	KHPATLRKYC	1.07	1.74	1.889	2
	128	137	KVVQPENLEY	1.066	1.79	2.087	3
	129	138	VVQPENLEYT	1.064	1.74	1.506	4
	86	95	QDKRFVCKHS	1.062	2.87	1.616	5
**AFN85178.1**				**Cut-off = 1.025**	**Cut-off = 1.317**	**Cut-off = 1**	
	243	252	PHAKKQDVVV	1.112	2.26	1.131	1
	51	60	KHPATLRKYC	1.07	1.74	1.873	2
	128	137	KVVQPENLEY	1.066	1.79	2.069	3
	129	138	VVQPENLEYT	1.064	1.74	1.493	4
	86	95	QDKRFVCKHS	1.062	2.87	1.601	5
**AOQ25641.1**				**Cut-off**	**Cut-off**	**Cut-off = 1**	

				**= 1.026**	**= 1.306**		
	243	252	PHAKKQDVVV	1.112	2.26	1.181	1
	51	60	KHPATLRKYC	1.07	1.74	1.955	2
	128	137	KVVQPENLEY	1.066	1.79	2.16	3
	129	138	VVQPENLEYT	1.064	1.74	1.559	4
	86	95	QDKRFVCKHS	1.062	2.87	1.672	5
**AOQ25658.1**				**Cut-off = 1.025**	**Cut-off = 1.289**	**Cut-off = 1**	
	243	252	PHAKKQDVVV	1.112	2.26	1.165	1
	51	60	KHPATLRKYC	1.07	1.74	1.929	2
	86	95	QDKRFVCKHS	1.062	2.87	1.649	3
	356	365	PIVTEKDSPV	1.061	2.4	1.197	4
	55	64	TLRKYCIEAK	1.053	1.3	1.113	5

Table S3. Antigenicity, surface accessibility, and hydrophilicity of epitopes of DENV-3 E protein.

**GenBank Accession No.**	**Position**			**Antigenicity**	**Hydrophilicity**	**Surface accessibility**	**Rank according to antigenicity**
**QXI72689.1**	**Start**	**End**	**Peptide Sequence**	**Cut-off = 1.025**	**Cut-off = 1.378**	**Cut-off = 1**	
	90	99	YVCKHTYVDR	1.118	1.74	1.042	1
	128	137	KVVQHENLKY	1.078	1.58	2.091	2
	129	138	VVQHENLKYT	1.076	1.53	1.509	3
	130	139	VQHENLKYTV	1.076	1.53	1.509	4
	354	363	PVVTKKEEPV	1.075	2.53	1.894	5
**QXI72690.1**				**Cut-off = 1.023**	**Cut-off = 1.405**	**Cut-off = 1**	
	90	99	YVCKHTYVDR	1.118	1.74	1.042	1
	128	137	KVVQHENLKY	1.078	1.58	2.09	2
	129	138	VVQHENLKYT	1.076	1.53	1.508	3
	130	139	VQHENLKYTV	1.076	1.53	1.508	4
	131	140	QHENLKYTVV	1.076	1.53	1.508	5
**AYP74620.1**				**Cut-off = 1.026**	**Cut-off = 1.387**	**Cut-off = 1**	

	90	99	YVCKHTYVDR	1.118	1.74	1.042	1
	128	137	KVVQHENLKY	1.078	1.58	2.09	2
	129	138	VVQHENLKYT	1.076	1.53	1.508	3
	130	139	VQHENLKYTV	1.076	1.53	1.508	4
	131	140	QHENLKYTVV	1.076	1.53	1.508	5
**AYP74622.1**				**Cut-off = 1.026**	**Cut-off = 1.369**	**Cut-off = 1**	
	90	99	YVCKHTYVDR	1.118	1.74	1.041	1
	128	137	KVVQHENLKY	1.078	1.58	2.089	2
	129	138	VVQHENLKYT	1.076	1.53	1.507	3
	130	139	VQHENLKYTV	1.076	1.53	1.507	4
	354	363	PVVTKKEEPV	1.075	2.53	1.892	5
**AFN85216.1**				**Cut-off = 1.025**	**Cut-off = 1.378**	**Cut-off = 1**	
	90	99	YVCKHTYVDR	1.118	1.74	1.042	1
	128	137	KVVQHENLKY	1.078	1.58	2.091	2
	129	138	VVQHENLKYT	1.076	1.53	1.509	3
	130	139	VQHENLKYTV	1.076	1.53	1.509	4
	354	363	PVVTKKEEPV	1.075	2.53	1.894	5
**ASV49464.1**				**Cut-off = 1.026**	**Cut-off = 1.372**	**Cut-off = 1**	
	90	99	YVCKHTYVDR	1.118	1.74	1.07	1
	128	137	KVVQHENLKY	1.078	1.58	2.147	2
	129	138	VVQHENLKYT	1.076	1.53	1.55	3
	130	139	VQHENLKYTV	1.076	1.53	1.55	4
	354	363	PVVTKKEEPV	1.075	2.53	1.945	5
**AOQ25535.1**				**Cut-off = 1.026**	**Cut-off = 1.366**	**Cut-off = 1**	
	90	99	YVCKHTYVDR	1.118	1.74	1.054	1
	128	137	KVVQHENLKY	1.078	1.58	2.114	2
	129	138	VVQHENLKYT	1.076	1.53	1.525	3
	130	139	VQHENLKYTV	1.076	1.53	1.525	4
	354	363	PVVTKKEEPV	1.075	2.53	1.914	5
**AOQ25562.1**				**Cut-off**	**Cut-off**	**Cut-off = 1**	

				= 1.026	= 1.364		
	90	99	YVCKHTYVDR	1.118	1.74	1.045	1
	354	363	PVVSKKEEPV	1.085	2.66	1.763	2
	128	137	KVVQHENLKY	1.078	1.58	2.096	3
	129	138	VVQHENLKYT	1.076	1.53	1.513	4
	130	139	VQHENLKYTV	1.076	1.53	1.513	5
**AOQ25777.1**				**Cut-off = 1.025**	**Cut-off = 1.385**	**Cut-off = 1**	
	90	99	YVCKHTYVDR	1.118	1.74	1.039	1
	128	137	KVVQHENLKY	1.078	1.58	2.085	2
	129	138	VVQHENLKYT	1.076	1.53	1.505	3
	130	139	VQHENLKYTV	1.076	1.53	1.505	4
	354	363	PVVTKKEEPV	1.075	2.53	1.889	5
**AFN85209.1**				**Cut-off = 1.026**	**Cut-off = 1.369**	**Cut-off = 1**	
	90	99	YVCKHTYVDR	1.118	1.74	1.052	1
	128	137	KVVQHENLKY	1.078	1.58	2.111	2
	129	138	VVQHENLKYT	1.076	1.53	1.524	3
	130	139	VQHENLKYTV	1.076	1.53	1.524	4
	354	363	PVVTKKEEPV	1.075	2.53	1.912	5

Table S4. Antigenicity, surface accessibility, and hydrophilicity of epitopes of DENV-4 E protein.

**GenBank Accession No.**	**Position**			**Antigenicity**	**Hydrophilicity**	**Surface accessibility**	**Rank according to antigenicity**
**KT750006.1**	**Start**	**End**	**Peptide Sequence**	**Cut-off = 1.027**	**Cut-off = 1.404**	**Cut-off = 1**	
	166	175	PRSPSVEVKL	1.082	1.83	1.545	1
	167	176	RSPSVEVKLP	1.082	1.83	1.545	2
	168	177	SPSVEVKLPE	1.08	2.19	1.366	3
	244	253	HAKRQDVTVL	1.078	1.87	1.192	4
	170	179	SVEVKLPEYG	1.076	1.71	1.022	5
**JN575591.1**				**Cut-off = 1.027**	**Cut-off = 1.418**	**Cut-off = 1**	
	169	178	PSVEVKLPDY	1.096	1.57	1.561	1

	166	175	PRSPSVEVKL	1.082	1.83	1.565	2
	167	176	RSPSVEVKLP	1.082	1.83	1.565	3
	168	177	SPSVEVKLPD	1.081	2.41	1.335	4
	244	253	HAKRQDVTVL	1.078	1.87	1.208	5
**U18435.1**				**Cut-off = 1.029**	**Cut-off = 1.206**	**Cut-off = 1**	
	219	228	PSVEVKLPDY	1.096	1.57	1.663	1
	216	225	PRSPSVEVKL	1.082	1.83	1.668	2
	217	226	RSPSVEVKLP	1.082	1.83	1.668	3
	366	375	QHGTTVVKVK	1.082	2.45	1.001	4
	218	227	SPSVEVKLPD	1.081	2.41	1.422	5
**KJ946244.1**				**Cut-off = 1.027**	**Cut-off = 1.445**	**Cut-off = 1**	
	169	178	PSVEVKLPDY	1.096	1.57	1.542	1
	166	175	PRSPSVEVKL	1.082	1.83	1.547	2
	167	176	RSPSVEVKLP	1.082	1.83	1.547	3
	168	177	SPSVEVKLPD	1.081	2.41	1.319	4
	244	253	HAKRQDVTVL	1.078	1.87	1.193	5
**EU448458.1**				**Cut-off = 1.027**	**Cut-off = 1.408**	**Cut-off = 1**	
	169	178	PSVEVKLPDY	1.096	1.57	1.541	1
	166	175	PRSPSVEVKL	1.082	1.83	1.546	2
	167	176	RSPSVEVKLP	1.082	1.83	1.546	3
	168	177	SPSVEVKLPD	1.081	2.41	1.318	4
	244	253	HAKRQDVTVL	1.078	1.87	1.193	5
**MG895393.1**				**Cut-off = 1.026**	**Cut-off = 1.426**	**Cut-off = 1**	
	169	178	PSVEVKLPDY	1.096	1.57	1.534	1
	166	175	PRSPSVEVKL	1.082	1.83	1.539	2
	167	176	RSPSVEVKLP	1.082	1.83	1.539	3
	168	177	SPSVEVKLPD	1.081	2.41	1.312	4
	244	253	HAKRQDVTVL	1.078	1.87	1.187	5
**BCG29769.1**				**Cut-off = 1.028**	**Cut-off = 1.361**	**Cut-off = 1**	

	169	178	PSVEVKLPDY	1.096	1.57	1.568	1
	167	176	RSPSVEVKLP	1.082	1.83	1.573	2
	166	175	PRSPSVEVKL	1.082	1.83	1.573	3
	168	177	SPSVEVKLPD	1.081	2.41	1.341	4
	244	253	HAKRQDVTVL	1.078	1.87	1.214	5
**ACC68759.1**				**Cut-off = 1.027**	**Cut-off = 1.408**	**Cut-off = 1**	
	169	178	PSVEVKLPDY	1.096	1.57	1.541	1
	166	175	PRSPSVEVKL	1.082	1.83	1.546	2
	167	176	RSPSVEVKLP	1.082	1.83	1.546	3
	168	177	SPSVEVKLPD	1.081	2.41	1.318	4
	244	253	HAKRQDVTVL	1.078	1.87	1.193	5
**AOQ25530.1**				**Cut-off = 1.027**	**Cut-off = 1.404**	**Cut-off = 1**	
	166	175	PRSPSVEVKL	1.082	1.83	1.545	1
	167	176	RSPSVEVKLP	1.082	1.83	1.545	2
	168	177	SPSVEVKLPE	1.08	2.19	1.366	3
	244	253	HAKRQDVTVL	1.078	1.87	1.192	4
	170	179	SVEVKLPEYG	1.076	1.71	1.022	5
**ANC57623.1**				**Cut-off = 1.027**	**Cut-off = 1.428**	**Cut-off = 1**	
	169	178	PSVEVKLPDY	1.096	1.57	1.511	1
	166	175	PRSPSVEVKL	1.082	1.83	1.516	2
	167	176	RSPSVEVKLP	1.082	1.83	1.516	3
	168	177	SPSVEVKLPD	1.081	2.41	1.292	4
	244	253	HAKRQDVTVL	1.078	1.87	1.169	5
